# TCF21 regulates miR-10a-5p/LIN28B signaling to block the proliferation and invasion of melanoma cells

**DOI:** 10.1371/journal.pone.0255971

**Published:** 2021-08-23

**Authors:** Haijun Zhu, Mengshi Kang, Xinping Bai

**Affiliations:** Department of Plastic Surgery, The Central Hospital of Wuhan, Wuhan, China; Children’s National Hospital, UNITED STATES

## Abstract

**Background and aim:**

Some research has suggested that miRNA-10a (miR-10a-5p) had an inhibitory function in proliferation and invasion of cancers. Whereas the role of miR-10a-5p in melanoma has not been fully explored. This study aims to confirm LIN28B as the targeted gene of miR-10a-5p which was explored in melanoma cells. In addition, upstream regulatory molecule of miR-10a-5p was also investigated in melanoma cells.

**Methods:**

Real-time Quantitative polymerase chain reaction (RT-qPCR) was adopted to analyze miR-10a-5p expression level in melanoma and the normal human epidermal melanocyte cells. Several biological assays were performed to evaluate miR-10a-5p influences on cell proliferation, migration and invasion ability in A375 and B16-F10 cells. Gene prediction of miRNA targeting and a dual luciferase assay were applied to assess miR-10a-5p-targeted LIN28B. Western blot assessed the impacts of miR-10a-5p on the protein expression of LIN28B. Western blot analyzed the TCF21 effects on the expression of LIN28B and RT-qPCR assessed the influence of TCF21 on the expression level of miRNA-10a. In addition, Chromatin Immunoprecipitation (ChIP) Assay and JASPAR databases were employed to explore the regulatory relationship between TCF21 and miR-10a-5p.

**Results:**

We discovered that miR-10a-5p expression was lower in melanoma cells and high expression of miR-10a-5p suppressed the proliferation, migration and invasion abilities of melanoma cells. We also discovered that miR-10a-5p targeted the LIN28B mRNA 3′UTR area and diminished LIN28B protein expression. We found that LIN28B expression was strongly decreased by TCF21 upregulation in the two melanoma cells. The qRT-PCR assay showed that miR-10a-5p expression level was obviously boosted by increased TCF21 expression. The results also demonstrated that TCF21 directly regulated miR-10a-5p at transcript levels.

**Conclusion:**

TCF21 induced miRNA-10a targeting LIN28B could affect the progression and growth of melanoma.

## Introduction

Melanoma is the most malignant skin carcinoma with steadily increasing incidence over the past three decades, responsible for the majority of skin cancer-associated deaths globally [1]. Melanoma arises from the melanocyte that can be located in the skin, mouth, eyes as well as intestinal tissues [2, 3]. Previous studies have revealed the critical issues for melanoma pathogenesis including genetic factors and ultraviolet radiation exposure [4]. Resistance, toxicity and incomplete therapeutic response for melanoma patients often rise despite the advanced melanoma has been considerably improved duo to the utilization of targeted therapy and immunotherapy [5, 6]. Thus, it is significant to further explore novel prevention strategies and therapeutic approaches for melanoma patients.

MicroRNAs (miRNAs) are a large group of short non-coding RNA molecules, which regulate gene expression at post-transcriptional level through directly binding with the 3’-untranslated region (UTR) of target genes [7], in some cases, microRNAs regulate gene expression also by targeting the 5 ’UTR [8]. MiRNAs have been revealed to be linked to a variety of biological processes such as cell proliferation, migration, inflammation and apoptosis [9, 10]. On the other hand, studies have illustrated that miRNAs functioned as cancer-promoting or a cancer-suppressive genes, which played essential roles in tumorigenesis [11–13], As for miRNAs, a number of studies showed the roles of miR-10a-5p on cancers [14, 15]. Otherwise, evidence has demonstrated that TF-miRNA axis played an important role in the development of carcinomas [16, 17]. In this present study, miR-10a-5p was significantly decreased in melanoma on the basis of bioinformatics methods. Whereas fewer studies have explored the effect of TF-miR-10a-5p on the progression of melanoma.

Here, we observed that miR-10a-5p attenuated the growth, invasion of melanoma by directly targeting and suppressing LIN28B expression. In addition, we verified that the TCF21/miR-10a-5p/LIN28B axis played a key role in proliferation, invasion, migration in melanoma. Collectively, our studies helped us understand the TF-miR-10a-5p mediated melanoma pathogenesis and may discover novel therapeutic targets for melanoma.

## Methods and materials

### Bioinformatics data analysis

Melanoma miRNA raw data as well as clinical information were retrieved from TCGA database (S1 Table). After this, DESeq2 package [18] was employed to conduct the normalization of the obtained melanoma miRNAs. Next, DESeq2 package [18] was applied to screen the differentially expressed miRNAs (DEMs) related to melanoma between the early-stage melanoma samples (TNM stage I-II, 231 samples) and the advanced stage samples (TNM stage III-IV, 207 samples) with P<0.05 and logFC>1.5 as the threshold. Then, melanoma DEMs were further selected across the overlapped analysis of miRNAs associated with overall survival (OS-related miRNAs). Afterwards, the overlapped miRNAs were observed by applying Venn diagram via vennDiagram package. Furthermore, the volcano map was performed employing ggplot package to compare and analyze the expression level of the determined DEMs.

### Identification of miR-10a-5p target genes

The four prediction tools for miRNA-mRNA association, including miRWalk2.0 [19], TargetScan6.2 [20], miRanda [21] and RNA22 [22] were applied to predict the candidate target genes of miR-10a-5p. Afterwards, the predicted target genes were made use of to perform overlapped genes assay with DEGs from GEO database (GSE31909 and GSE35388 sets). The DEGs between the melanoma samples and the non-melanoma samples were assessed based on the above selected method [23]. Furthermore, PCR assay and western blot assay were carried on confirming the result.

### Cell culture and transfection

The melanoma cell lines A375 (human), B16-F10 (mouse) and the normal human epidermal melanocyte HeMa-Lp were achieved from the American Type Culture Collection (Manassas, VA). In addition, mouse melanocytes (melan-a) were established in Alpaca Biological Engineering Laboratory, Shanxi Agricultural University, Taigu, China. Melanocytes were cultured in MelM (ScienCell, Carlsbad, CA, USA). The total cells were maintained in DMEM (Thermo Fisher Scientific, Inc.) supplemented with 10% FBS (Thermo Fisher Scientific, Inc.) and 1% streptomycin-penicillin in a humidified chamber with 5% CO2 at 37°C.

The miR-10a-5p mimic, the mimics negative control (miR-NC) and miR-10a-5p inhibitor were produced and supplied by GenePharma (Shanghai, China). The pcDNA (Vector) and pcDNA-LIN28B overexpression (LIN28B-OE) plasmids were designed by GenePharma (Shanghai, China). The pcDNA (Vector) and pcDNA-TCF21 overexpression (TCF21-OE) plasmids were designed by GenePharma (Shanghai, China). The miR-10a-5p mimic (50 nM), miR-10a-5p inhibitors (50 nM) as well as recombinant plasmids (50 nM) were introduced into the melanoma cells by employing Lipofectamine 2000 (Invitrogen) on the basis of the manufacturer’s protocols. Finally, the transfection efficiency was assessed according to qRT-PCR assay after 48 h of transfection at 37°C.

### Western blot analysis

Cell lysis buffer (Thermo Fisher Scientific Inc., Rockford, IL, USA) was utilized to extract total proteins from target cells for western blotting assay in accordance to the manufacturer’s protocols. The protein level of each sample was quantified via the BCA kit (Pierce, USA). Next, the proteins (30μg) were separated through 10% SDS polyacrylamide gels (SDS-PAGE) and then transferred to polyvinylidene fluoride (PVDF) membranes at 100 V for 2.5 h. After that, the membrane was blocked by employing 5% fat-free milk for 1 h, followed by incubated with primary antibody (β-actin, 1:200, ab115777, Abcam; LIN28B, 1:2000, ab191881, Abcam, for both melanoma cell lines A375 (human), B16-F10 (mouse); TCF21, 1:3000, ab182134, Abcam) overnight at 4°C. The next day, the membrane was washed for 3 times with TBST and then incubated with secondary antibody goat anti-rabbit HRP-linked IgG antibody 1:2000, (Cell Signaling, Danvers, MA, USA). Finally, the outcomes were visualized via chemiluminescence (Millipore, MA, USA).

### Quantitative reverse transcription-polymerase chain reaction (qRT-PCR)

To test the relative expression levels of miRNA and mRNAs, total RNA was extracted by applying a miRNeasy mini kit (Qiagen, Hilden, Germany) on the basis of the manufacturer’s protocols. A260/280 was used to assess RNA quality and the elution volume was 20 ul during RNA extraction. Next, 75ng of RNA was reversely transcribed into complementary DNA (cDNA) by using Quant cDNA first strand synthesis kits (Tiangen Biotech) The reaction, run at 37°C for 15 min, 85°C for 5 secs, and then was saved at 4°C. RT-qPCR was carried out in triplicates via an ABI 7500 instrument (7500, Applied Biosystems, Foster City, CA). The qPCR was performed as following condition: at 94°C for 2 minutes, 10 cycles with each at 94°C for 10 secs, 30 secs at 55°C, 2.5 min at 68°C, followed by 25 cycles with each 10 secs at 94°C, 25 at 55°C cycles for 30 secs, 68°C for 2.5 min, a final extension step was conducted at 60°C for 10 min. The fold changes were assessed by using relative quantification (2^−ΔΔCT^ approach). U6 was used as housekeeping for miRNA expression normalization, while 18S rRNA was used for mRNA expression normalization. The primers applied for qRT-PCR were listed in Table 1.

**Table 1:** All special primers were listed in this study.

Primer	Sequence
MiR-10a-5p	Forward: 5’-TGCGGTACCCTGTAGATCCG-3′
	Reverse: -5′-CCAGTGCAGGGTCCGAGGT-3′
U6	Forward: 5’-CTCGCTTCGGCAGCACA-3’
	Reverse: 5’-AACGCTTCACGAATTTGCGT-3’
18s RNA	Forward: 5′-GATGGTAGTCGCCGTGCC-3′
	Reverse: 5′-GCCTGCTGCCTTCCTTGG-3′
MiR-651	Forward: 5′-CGCAGTTTAGGATAAGCTTG-3′
	Reverse: 5′-TCCAGTTTTTTTTTTTTTTTCAAAAGTC-3′
MiR-2110	Forward: 5′-GGAAACGGCCGCTGA-3′
MiR-29c	Reverse: 5′-GGTCCAGTTTTTTTTTTTTTTTCACT-3′
MiR-146b	Forward: 5′-ACACTCCAGCTGGGTGACCGATTTCTCCTC-3′
MiR-320c-1	Reverse: 5′-TGGTGTCGTG GAGTCG-3′
MiR-3691	Forward: 5′-TGACCCATCCTGGGCCTCAA-3′
MiR-3682	Reverse: 5′-CCAGTGGGCAAGATGTGGGCC-3′
LIN28B	Forward: 5′-CAGAAAAGCTGGGTTGAGA-3′
FBXO22	Reverse: 5′-GTCCAGTTTTTTTTTTTTTTTACCCT-3′
UAP1	Forward: 5′-GCAGAGTGGATGATGGAGAC-3′
	Reverse: 5′-CCAGTTTTTTTTTTTTTTTGTACCGA-3′
TCF21	Forward: 5′-GCAGCTACTTCTACCTGTGT-3′
	Reverse: 5′-CAGGTCCAGTTTTTTTTTTTTTTTATGA-3′
CDK6	Forward: 5′-GTCAATACGGGTAACAGGAC-3′
MAZ	Reverse: 5′-TTCTTTGGCTGAGGAGGTAG-3′’
	Forward: 5′-CGGAGCACCTTCGTGTTGA-3′
	Reverse: 5′-CACACACTCCCTCCATAAGCG-3′
ZNF148	Forward: 5’-TTGCATTCAGAAAGGAGCAGACT-3’;
	Reverse: 5’-CAACTGGTTCTGTAGGGTTCGTTT-3’
LEF1	Forward: 5′-GCAGATCCTGGCTAACGACA-3′
	Reverse: 5′-GTAAAGTGTTCTCGCGGGGT-3′
	Forward: 5′-CGGGATCCACCATGGAGAAGGACGGCCTG-3′
	Reverse: 5′-CGGATCCATTGCTCAGGCTGTATTCAGCTCCGA-3′
	Forward: 5’-CTAACGGGATCCATGTTCCCGGTGTTTCCTTGCACGCTGC-3’
	Reverse: 5’-CTAACGGAATTCTCACCAGGGTTGGGAGGGA AGTGGC-3’
	Forward: 5’-TGATGATGCCATGCAGTTTT-3’
	Reverse: 5’-TCCCTGCTGTTGTTACT TGCT-3’
	Forward: 5’-AGAACACCCCGATGACGGA-3’
	Reverse: 5’-GGCATCATTATGTACCCGGAAT-3’

### Luciferase reporter assay

Dual-Luciferase reporter assay was conducted to confirm the binding of miR-10a-5p with the 3’-UTR region of LIN28B in A375 and B16-F10 cells. Co-transfection of cells was performed using Lipofectamine 2000 reagent (Invitrogen) according to manufacturer protocol through a mixture of luciferase vector pmirGLO (Promega, Madison, Wisconsin, USA) containing predicted wild type or mutant binding sequences (Wt-LIN28B-3’UTR or mut-LIN28B-3’UTR) of LIN28B and nucleic acid fragments (miR-10a-5p mimic, miR-10a-5p inhibitor or NC mimic, GenePharma Shanghai, China). Finally, luciferase activities were determined with the Dual-Luciferase Reporter Assay System based on manufacturer’s instructions (Promega). The total transfections were conducted in duplicate.

### Transwell assay

The cells in serum-free medium were placed into upper chamber. DMEM containing 30% FBS was added into lower chamber. After 48 h of incubation, cells in the upper chamber were removed, whereas invasive cells were fixed, stained, and counted by using a microscope.

### 3-(4,5-Dimethyl-2-Thiazolyl)-2,5-Diphenyl-2-H-Tetrazolium Bromide (MTT) assay

The cell proliferation was tested via MTT assay. After transfection for 48 h, cells were seeded and incubated in 96-well plates at a density of 5000 cells/well for 4 h at 37°C. Briefly, each well was added with 10 μL of MTT solution (5 mg/mL) at 1, 2, 3, 4, 5 days, respectively for 4 h, and then was then dissolved with 200 μL of 150μl DMSO (Sigma). Finally, the optical density (OD) value at 570 nm of each well was assessed through a microplate reader. MTT assay was repeated for three times.

### Chromatin Immunoprecipitation (ChIP) assay

ChIP assay was carried out applying a ChIP Kit (Abcam) on the basis of manufacturer’s instructions. Briefly, cells were crosslinked with 1% formaldehyde for 10 minutes at room temperature and followed by sonification to remove the insoluble material. Immunoprecipitation was implemented by using the anti-TCF21 antibody (TCF21, 1:70, ab182134, Abcam) or the control IgG. The purified DNA was assessed via RT-qPCR using the specific primers. The primers for ChIP-qPCR analysis were exhibited in Table 1.

### Statistical analysis

Data were presented as the mean ± one standard deviation. The Student’s test, one-way analysis of variance was applied to compare between groups as appropriate. The Kaplan–Meier approach was employed to assess overall survival (OS), The median value was used as the cut-off to define high or low expression group. All statistical analyses were performed using GraphPad Prism 7.0 software. Differences were considered to be statistically significant at p < 0.05.

## Result

### MiR-10a-5p expression was reduced and correlated with longer OS in melanoma

**Fig 1:**
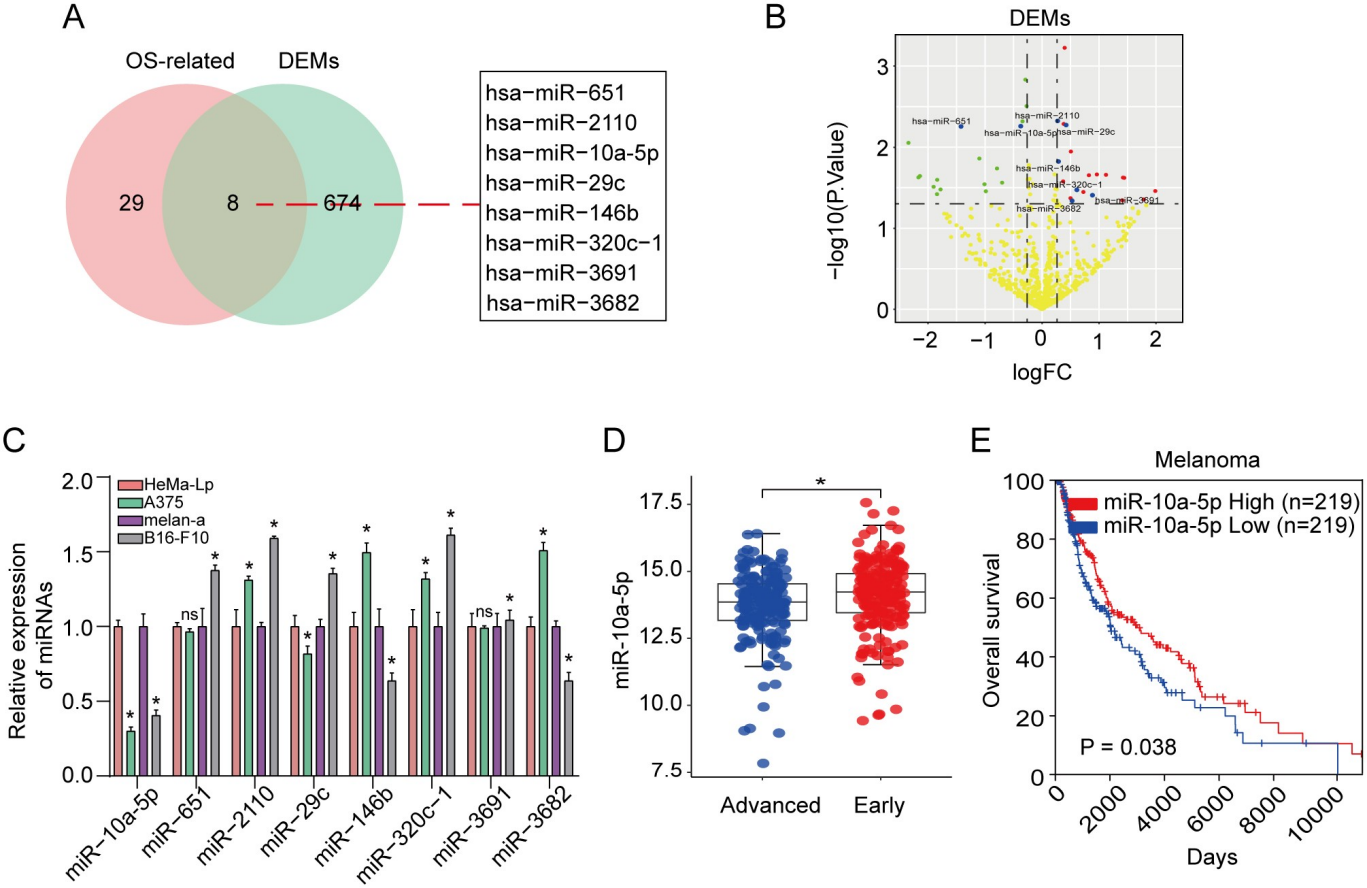
MiR-10a-5p expression was reduced and miR-10a-5p overexpression was correlated with longer OS in melanoma. **(A)** Eight DEMs were identified based on the overlapped analysis of melanoma miRNAs in the TCGA-SKCM between early-stage melanoma tissues, advanced stage tissues dataset and OS-correlated miRNAs. **(B)** The volcano plot of the eight DEMs selected from TCGA-SKCM dataset and OS-correlated miRNAs. Read and green balls represent elevated and decreased DEMs, respectively. Blue balls are screened 8 DEMs. **(C)** qRT-PCR assay was used to assess the expression of the eight DEMsin A375, B16-F10, HeMa-Lp and melan-a cell lines. **(D)** The comparison for miR-10a-5p expression was performed between early-stage melanoma and advanced stage melanoma in TCGA database. **(E)** Log-rank test for survival analysis was performed based on miR-10a-5p expression alteration. The study was repeated for three times. *P<0.05, **P<0.01.

Based on the overlapped analysis of DEMs in the TCGA-SKCM dataset and OS-correlated miRNAs (S1 Table), we discovered eight DEMs (Fig 1A) between early-stage melanoma tissues and advanced stage tissues. The volcano plot of the eight DEMs were exhibited in Fig 1B. We next detected the relative expression level of the above eight DEMs in melanoma cell lines and corresponding normal cells using RT-qPCR assay due to consensus alteration of the eight miRNAs in DEMs and OS-correlated miRNAs sets. We experimentally identified that miR-10a-5p expression was remarkably decreased in melanoma cells in comparison to corresponding normal cells via RT-qPCR (Fig 1C). The result of Fig 1D indicated that miR-10a-5p expression was higher in early-stage melanoma than that in advanced stage melanoma. After that, the relation between miR-10a-5p expression and prognosis of melanoma patients was explored by Kaplan-Meier survival assay. The results of Kaplan-Meier assay indicated that melanoma patients with low expression of miR-10a-5p had a poorer OS (P < 0.05) (Fig 1E, S2 Table). Together these results indicated that miR-10a-5p may function to inhibit melanoma development or progression.

### MiR-10a-5p attenuates proliferation, invasion and migration of A375 and B16-F10 cells

**Fig 2:**
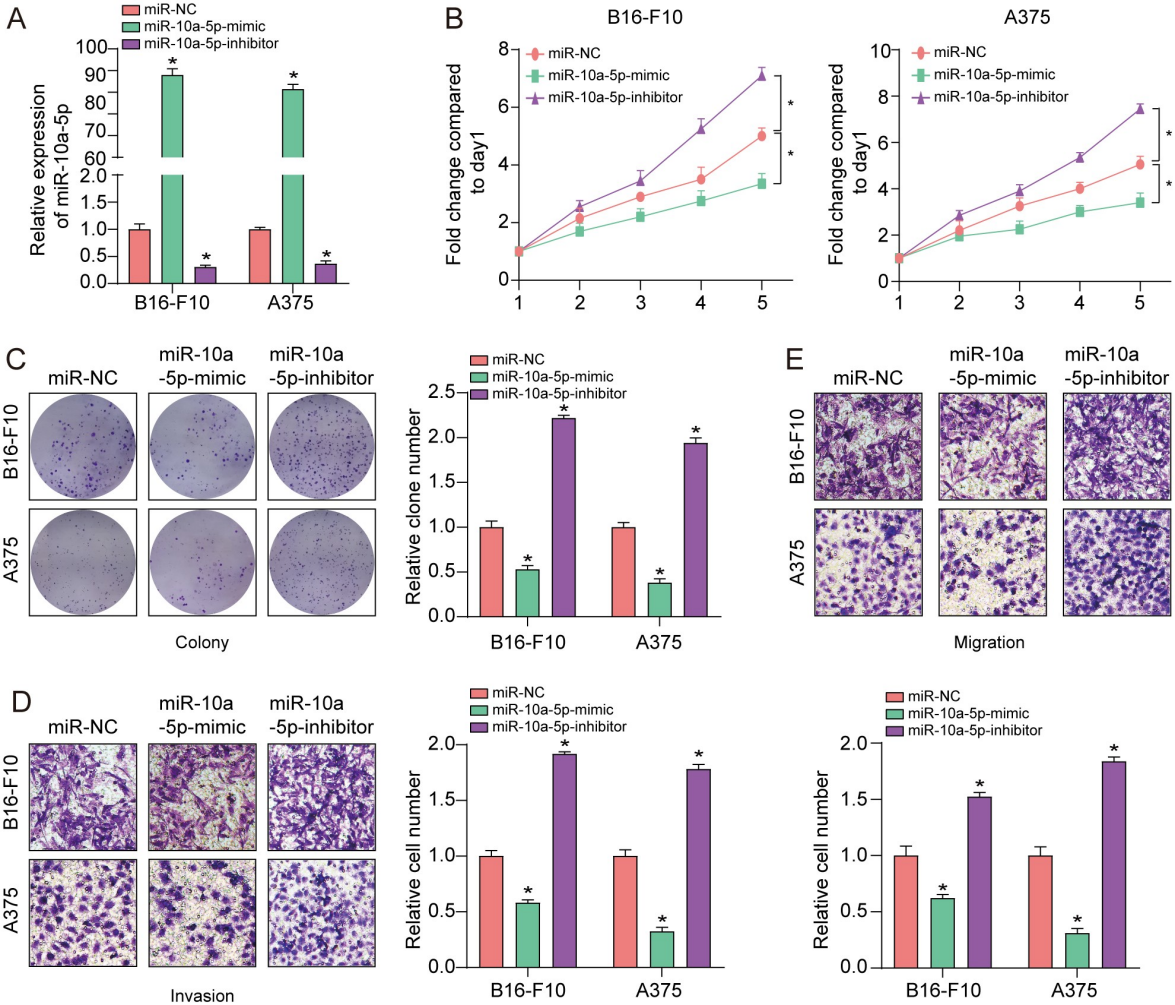
MiR-10a-5p attenuates proliferation, invasion and migration of melanoma cells. **(A)** qRT-PCR assay was applied to evaluate miR-10a-5p expression with miR-10a-5p mimic or miR-10a-5p inhibitor in A375 and B16-F10 cells. **(B & C)** The proliferative ability was evaluated across cell viability as well as colony formation with miR-10a-5p mimic or miR-10a-5p inhibitor in A375 and B16-F10 cells. **(D**, **E)** Transwell assay was employed to evaluate melanoma cell invasion and migration with miR-10a-5p mimic or miR-10a-5p inhibitor in A375 and B16-F10 cells. *P<0.05, **P<0.01. The study was repeated for three times.

To determine the role and function of miRNA-10a in melanoma cells, we developed miR-10a-5p overexpressed cells by transfection of miR-10a-5p mimic into melanoma cells and miR-10a-5p low-expressed cells via transfection of miR-10a-5p inhibitor into melanoma cells, respectively. The qRT-PCR result showed that transfection of miR-10a-5p mimic could obviously increase the expression of miR-10a-5p while transfection of miR-10a-5p inhibitor resulted in opposite results in the two melanoma cells (Fig 2A). As expected, overexpression of miR-10a-5p clearly impaired the proliferation, invasion and migration capability of melanoma cells. Whereas a diminished expression of miR-10a-5p could augment the proliferation, invasion and migration ability of melanoma cells in A375 and B16-F10 cells (Fig 2B–2E). These findings revealed that miR-10a-5p over-expression could impede melanoma growth and progression.

### LIN28B is a direct target of miR-10a-5p in melanoma cells

**Fig 3:**
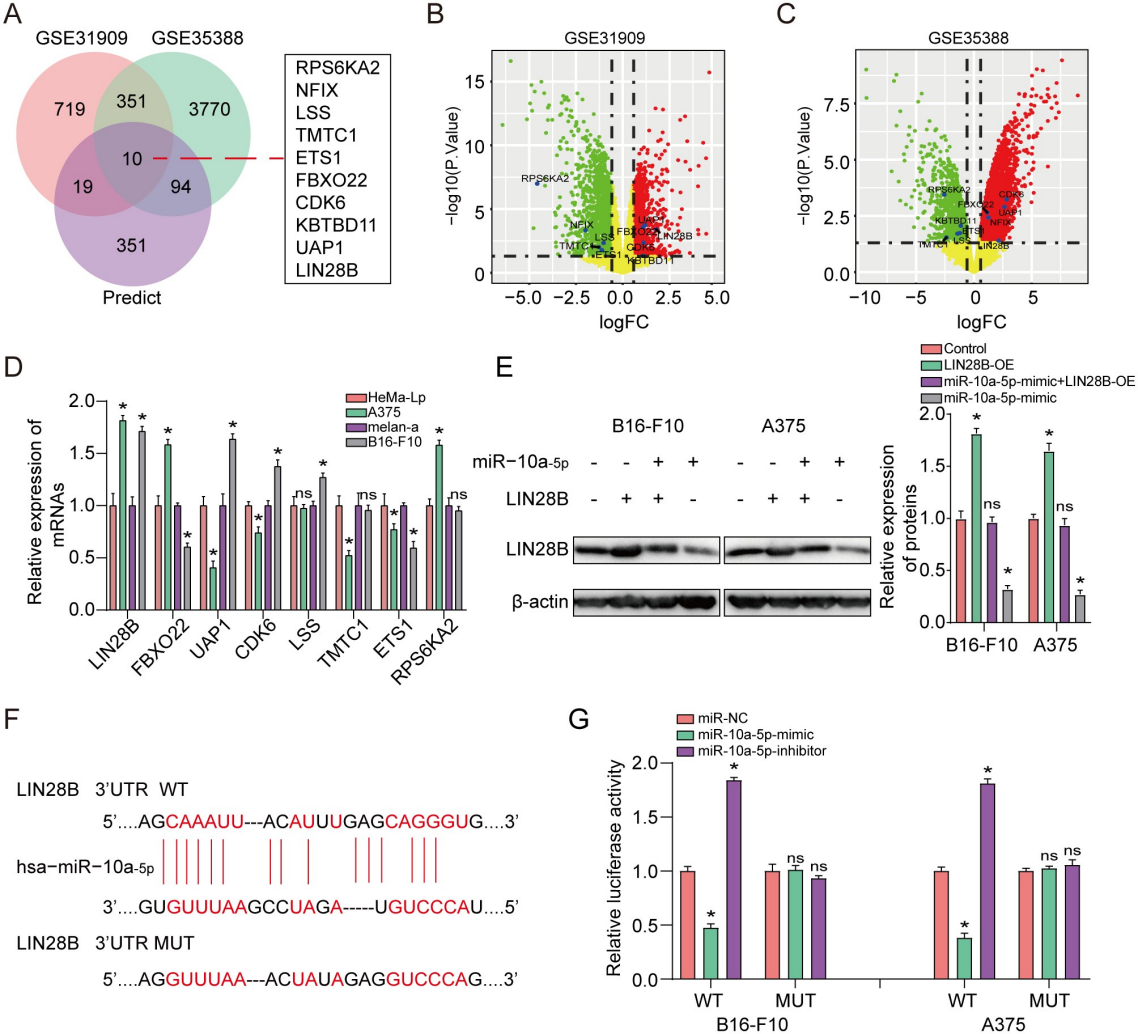
LIN28B is a direct target of miR-10a-5p in melanoma cells. **(A)** 10 common target genes were determined through DEGs’ overlapping analysis among GSE31909 set, GSE35388 set and the predicted target genes. **(B, C)** Volcano plot of the selected 10 DEGs in GSE31909 and GSE35388 sets, respectively. **(D)** QRT-PCR analysis for LIN28B, FBXO22, UAP1 and CDK6 in A375, B16-F10, HeMa-Lp and melan-a cells. **(E)** Western blot assay was used to assess the alteration of LIN28B with miR-10a-5p mimic. **(F)** The starbase database was applied to perform the bioinformatic analysis between miR-10a-5p and LIN28BMiR-10a-5p. **(G)** pmirGLO-REPORT luciferase vector consisting of LIN28B 3’UTR or a mutated type was co-transfected in A375 and B16-F10 cells with miR-10a-5p increase or decline or miR-NC. Firefly luciferase activity was evaluated via Renilla luciferase activity. *P<0.05, **P<0.01. The study was repeated for three times.

**Fig 4:**
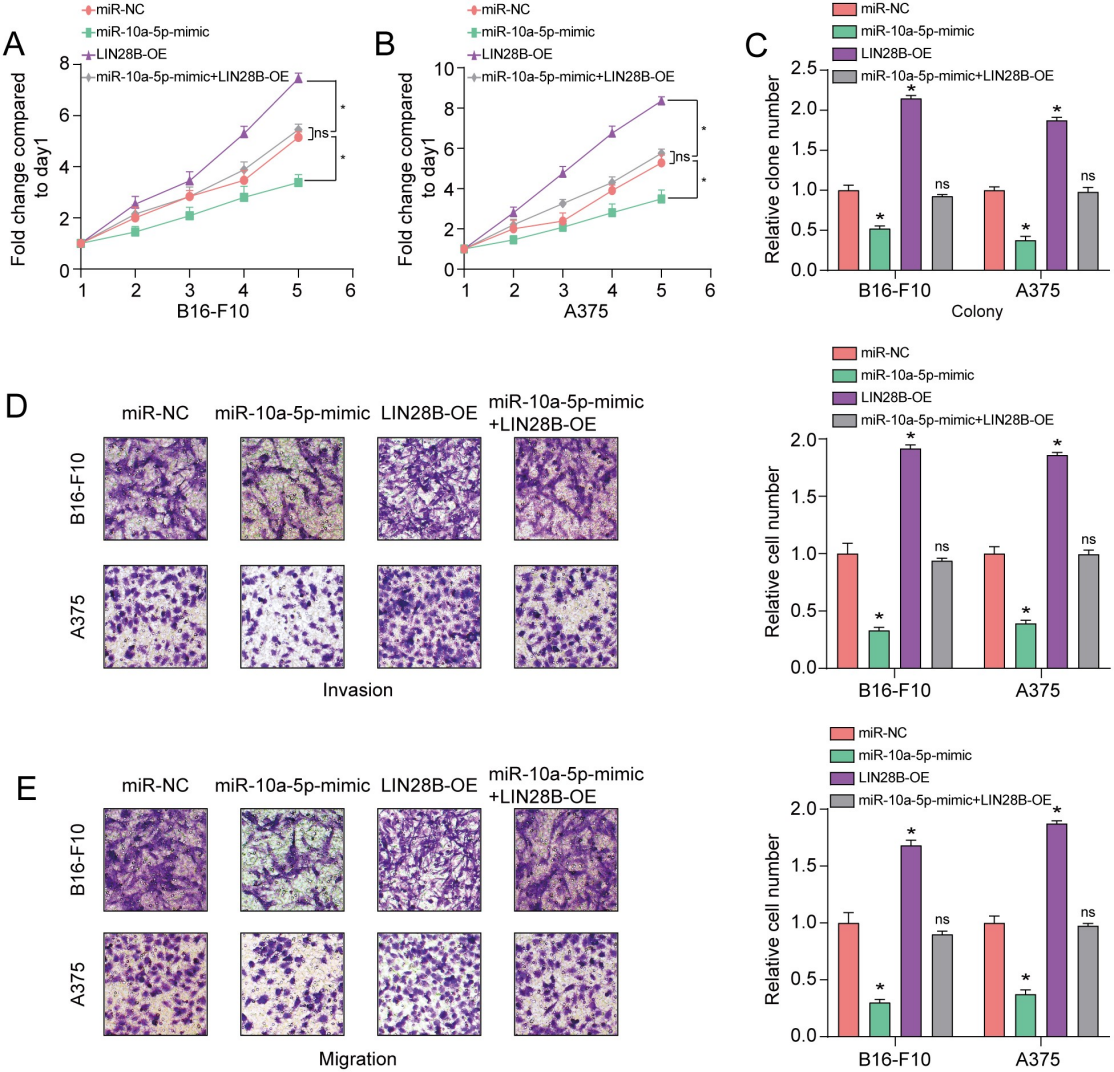
Restoration of LIN28B reverses the suppressive influence of miR-10a-5p in melanoma cells. **(A-C)** The influence of miR-10a-5p and LIN28B on the proliferative power of A375 and B16-F10 cells was measured through cell viability and colony formation. **(D, E)** The effect of miR-10a-5p and LIN28B on the cell invasion and migration capacity of A375 and B16-F10 cells was measured by using transwell assay. *P<0.05, **P<0.01. The study was repeated for three times.

To search for the potential target of miR-10a-5p, we first performed DEGs’ overlapping analysis among GSE31909 set and GSE35388 set and the predicted target genes (Fig 3A). The volcano plot of 10 DEGs were observed in GSE31909 and GSE35388 sets, respectively (Fig 3B & 3C). Importantly, as for the four intersected target genes, LIN28B was most significantly raised in melanoma cells in comparison to that in normal controls (Fig 3D). Then we measured the influence of miR-10a-5p alteration on LIN28B based on western blot analysis. The western blot assay result displayed that high miR-10a-5p expression remarkably reduced the protein levels of LIN28B in A375 and B16-F10 cells, whereas the decline of miR-10a-5p expression had a reverse impact (Fig 3E). As illustrated in Fig 3F, a putative binding site for miRNA-10a-5p was determined in the 3′UTR of LIN28B mRNA. In addition, a previous study also revealed LIN28B as a target of miR-10a-5p in human neural progenitor cells [24]. Then, dual-luciferase reporter assay was employed to verify the target association between miR-10a-5p and LIN28B. As shown in Fig 3G, increased miR-10a-5p expression obviously diminished the luciferase activity of the LIN28B-WT-3′-UTR plasmid in A375 and B16-F10 cell lines while decreased miR-10a-5p expression resulted in a reverse effect. Otherwise, the two melanoma cells with miR-10a-5p over-expression or low-expression showed no significant changes in the luciferase intensity of LIN28B-MUT-3’-UTR plasmid. The abovementioned results revealed a direct target association between miR-10a-5p and LIN28B. In addition, the result also displayed that elevated miR-10a-5p expression blocked the proliferation, invasion and migration of in the both melanoma cells and this effect could be reserved by high LIN28B expression (Fig 4A–4E). These results displayed that miR-10a-5p impaired LIN28B expression by directly targeting to 3′UTR of LIN28B mRNA in melanoma.

### TCF21 transcriptionally augmented miR-10a-5p expression

**Fig 5:**
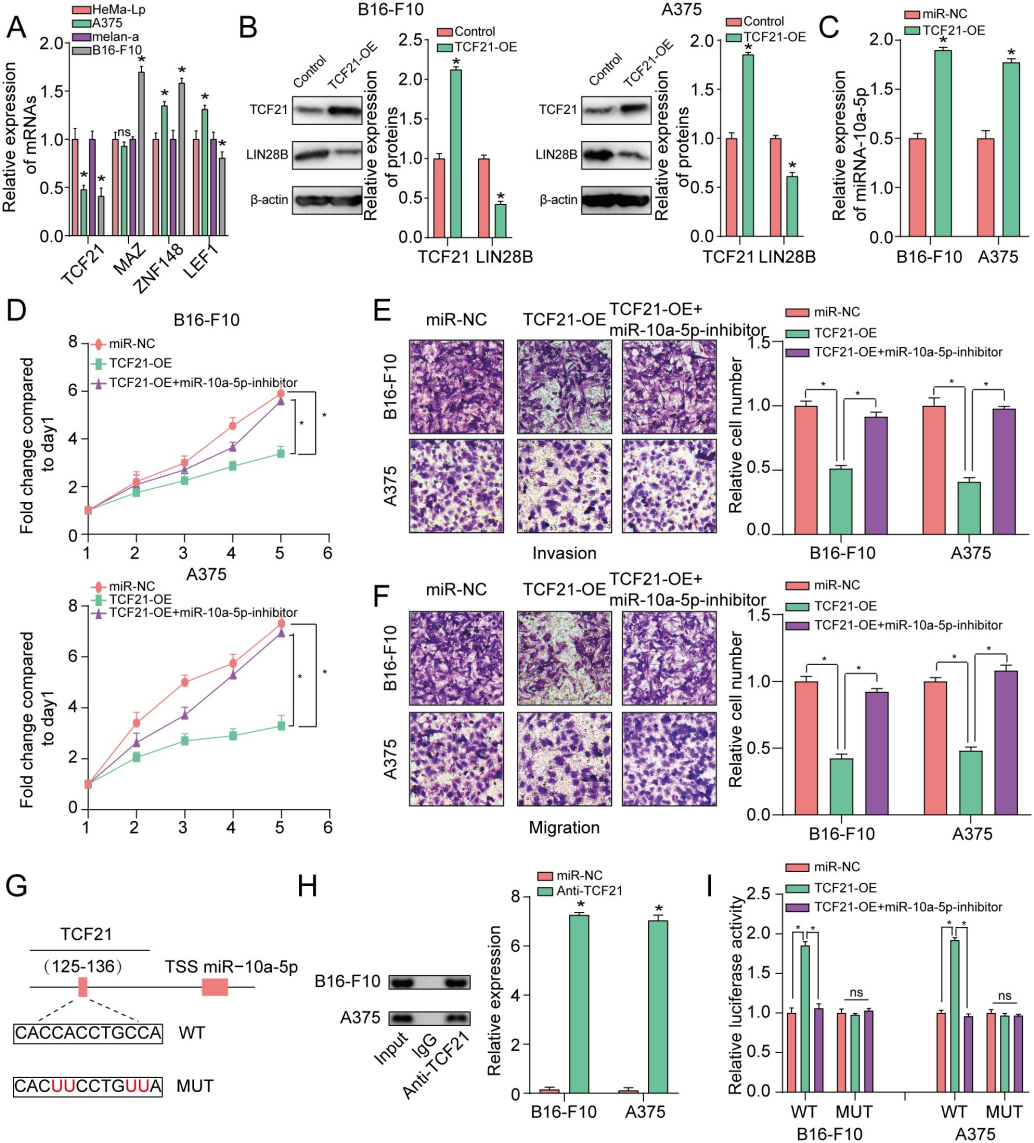
TCF21 transcriptionally augmented miR-10a-5p expression. **(A)** qRT-PCR assay for TCF21, MAZ, ZNF148 and LEF1 expression level. **(B)** The effect of TCF21 high expression on LIN28B in A375 and B16-F10 cells was assessed across western blot assay. **(C)** The effect of TCF21 high expression on miR-10a-5p expression in A375 and B16-F10 cells was analyzed by qRT-PCR assay. **(D)** High TCF21 expression significantly raised the number of melanoma-derived cell colonies formed via colony formation assay, and this effect was reversed by miR-10a-5p inhibitor. **(E, F)** Elevated TCF21 expression significantly inhibited migration and invasion of A375 and B16-F10 cells, which was rescued by miR-10a-5p inhibitor based on transwell assay. **(G)** A schematic illustrated the proximal region of the miR-10a-5p promoter that was analyzed by applying ChIP assay. **(H)** ChIP was adopted to verify the binding association between TCF21 and the miR-10a-5p promoter in A375 and B16-F10 cells. **(I)** Luciferase activity was promoted in the WT miR-10a-5p promoter with TCF21 increase that was significantly reversed by miR-10a-5p inhibitor. No significant alteration was found in luciferase intensity when TCF21 targeting site at 125-136bp were mutated. *P<0.05, **P<0.01. The study was repeated for three times.

Firstly, JASPAR databases (http://jaspar.genereg.net/) were applied to explore and analyze the putative TF binding sites for miR-10a-5p’ promoters. We next performed qRT-PCR assay to assess the expression level of the top four candidate TFs. These qRT-PCR assay results demonstrated that TCF21 was the most obviously diminished molecule in A375 and B16-F10 cells compared with HeMa-Lp and melan-a cell (Fig 5A). Then, we assessed LIN28B protein expression upon TCF21 high expression via western blot analysis. The result displayed that LIN28B expression were significantly diminished by TCF21 upregulation in the two melanoma cells (Fig 5B). The qRT-PCR assay findings showed that miR-10a-5p expression level was clearly boosted by increased TCF21 expression (Fig 5C). We further found that raised TCF21 expression could block melanoma cells’ proliferation, invasion and migration, which could be obviously reversed by decreased miR-10a-5p expression (Fig 5D–5F).

We then explored whether TCF21 regulated miR-10a-5p expression transcriptionally according to ChIP assay. Binding sites of TCF21 inside the putative miR-10a-5p promoter region was unveiled on the basis of the JASPAR database. As exhibited in Fig 5G, ChIP assay results displayed that the region at 125-136bp upstream of the pre-miR-10a-5p’ promoter region was directly targeted by TCF21. In addition, luciferase activity in the WT miR-10a-5p promoter was obviously strengthened under high TCF21 expression, which was prominently rescued by lessened miR-10a-5p expression. No significant alteration was found in luciferase intensity when TCF21 targeting site at 125-136bp were mutated (Fig 5H and 5I). These results suggested that that TCF21 directly regulated miR-10a-5p at transcript levels. The abovementioned findings showed that TCF21 induced miR-10a-5p blocked melanoma progression via directly targeting LIN28B.

## Discussion

Numerous studies have suggested that miRNAs played critical role in the growth and progression of different carcinomas via targeting tumor oncogenes or suppressor gene [25, 26]. Some studies also indicated that miRNAs were closely correlated with melanoma [27, 28], while the precise functions and molecular mechanisms of miRNAs in the initiation and progression of melanoma needs further exploration. The effect of miR-10a-5p on cancer development has been reported in various studies [29, 30]. A previous study indicated that miR-10a-5p was upregulated during early progression of melanoma [31]. Whereas its role and mechanism in melanoma is still unclear. In our study, we found that miR-10a-5p was obviously diminished in melanoma tissues by bioinformatics method, showing that miR-10a-5p serves as a tumor suppressor gene in melanoma.

The reduced miR-10a-5p expression has been found in different kinds of cancers. For example, Mu et al. demonstrated that miR-10a-5p served as a tumor suppressor in prostate cancer by targeting KDM4A [32]. Khan et al. found that miR-10a-5p was reduced in breast cancer [33], which were consistent with our findings. In our study, low miR-10a-5p expression was also discovered to attenuate the proliferation, invasion, migration of melanoma cells. On the other hand, Lovnicki et al. found that LIN28B promoted the development of neuroendocrine prostate cancer [34]. A previous study indicated that pancreatic circulating tumor cell profiling identified LIN28B as a metastasis driver and drug target [35]. Pang et al. showed that LIN28B promoted colon cancer migration and recurrence [36], which were also consistent with our findings. Furthermore, the data in our study suggested that LIN28B served as a direct target of miR-10a-5p in melanoma cells.

Various studies demonstrated that TFs played crucial roles in different tumors. For example, Malik et al. suggested that the TF CBFB suppressed breast cancer by orchestrating translation and transcription [37]. Chai et al. identified targeting TF STAT3 for cancer prevention and therapy [38]. In our study, TFs that targeted miR-10a-5p’ promoters were predicted on the basis of JASPAR database. Consequently, TCF21 was further determined as a key upstream regulatory molecule according to qRT-PCR assay. After that, we measured the influence of TCF21 high expression on LIN28B expression based on western blot analysis and miR-10a-5p expression by qRT-PCR assay. In addition, the effect of TCF21 high expression on proliferation, invasion and migration capacity of melanoma cells was also explored. Collectively, these results implied that TCF21/miR-10a-5p/LIN28B axis played a crucial role in the growth and invasion of melanoma. To conclude, miR-10a-5p was revealed to be highly expressed in melanoma tissues as well as cell lines, suppressing proliferation and progression of melanoma cells by directly regulating and blocking the expression of LIN28B. In addition, our study confirmed that TCF21 served as a key upstream regulatory molecule of miR-10a-5p.

## Supporting information

S1 Table The detailed clinical information of enrolled melanoma samples and the corresponding miRNA expression matrix. (CSV)

S2 Table The significance of the relationship between miRNA and prognosis. (CSV)

S1 Fig (TIF)

S2 Fig (TIF)
