# Supplementary figures and images for: TCF21 regulates miR-10a-5p/LIN28B signaling to block the proliferation and invasion of melanoma cells
Source: PLoS One. 2021 Aug 23;16(8):e0255971. doi: 10.1371/journal.pone.0255971 (PMC8382182; doi:10.1371/journal.pone.0255971)

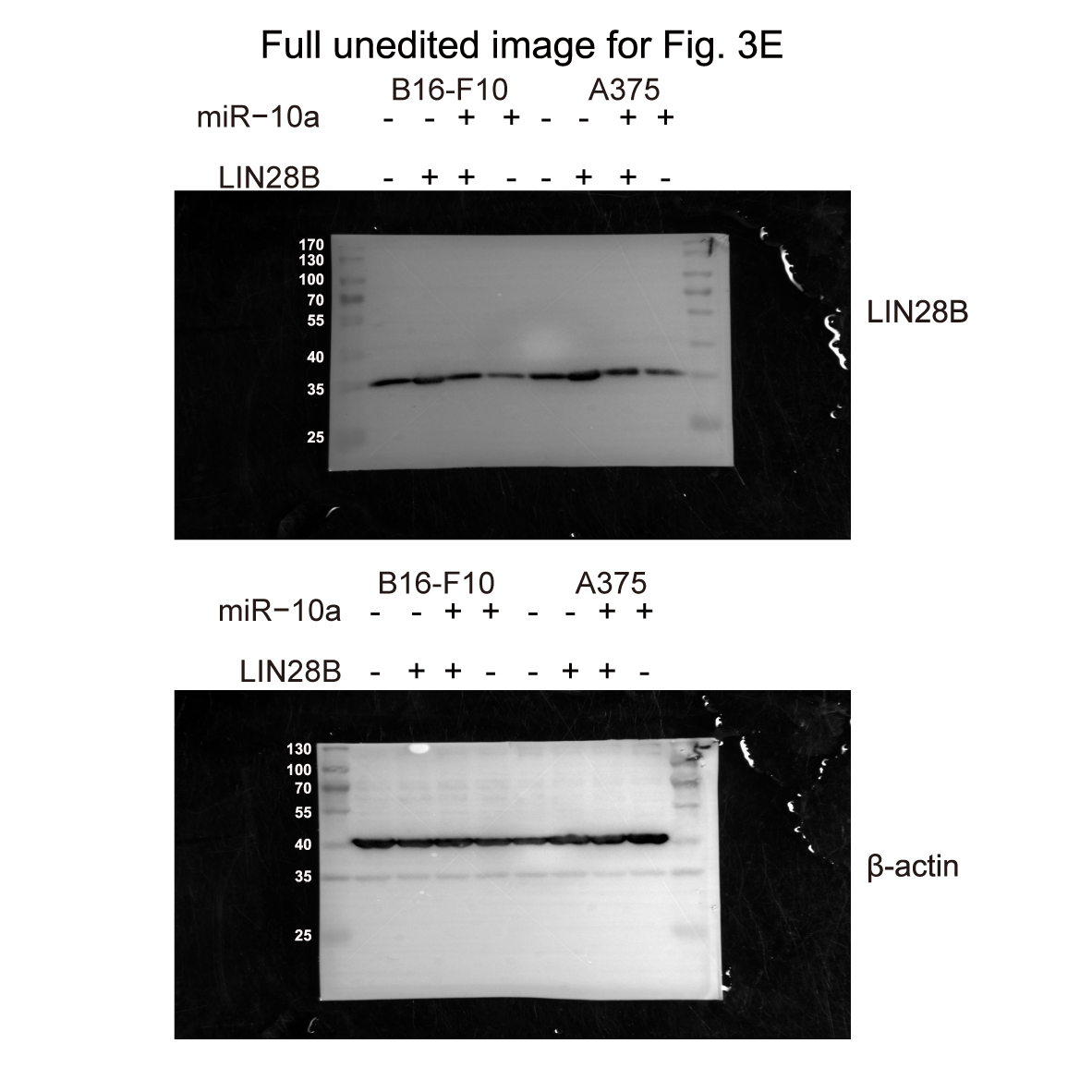

Supplement: S1 Fig — (TIF) [file pone.0255971.s003.tif]

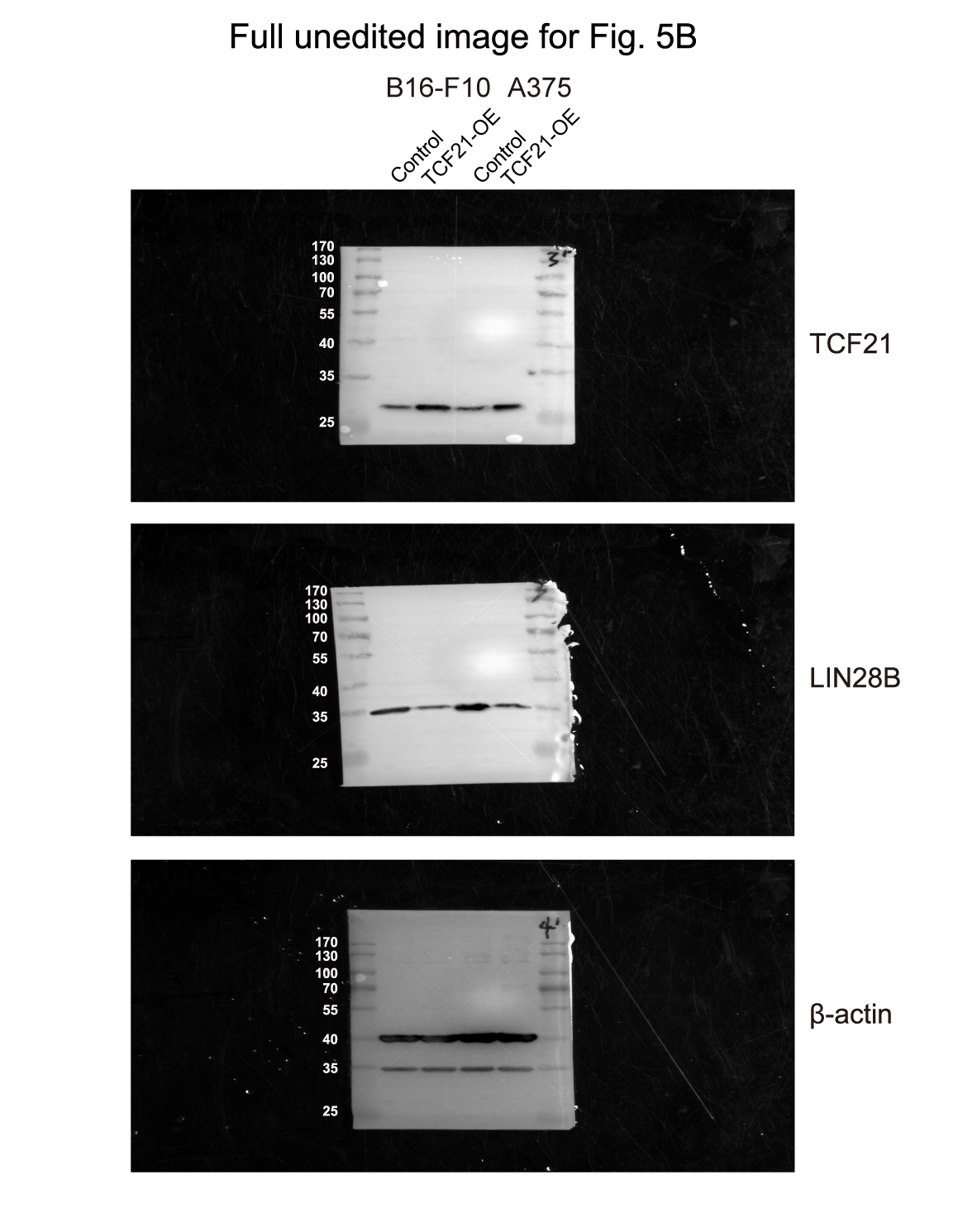

Supplement: S2 Fig — (TIF) [file pone.0255971.s004.tif]
